# Supplementary material for: Role of Phosphodiesterase 1 in the Regulation of Real-Time cGMP Levels and Contractility in Adult Mouse Cardiomyocytes
Source: Cells. 2023 Dec 3;12(23):2759. doi: 10.3390/cells12232759 (PMC10706287; doi:10.3390/cells12232759)
Supplement: Supplementary file 1 [file cells-12-02759-s001.zip › cells-2615308-supplementary.pdf]

Supplementary Figure S1

(**A**)

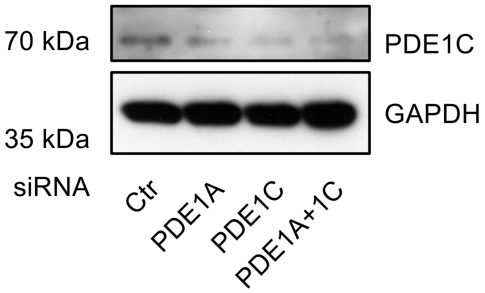

(**B**)

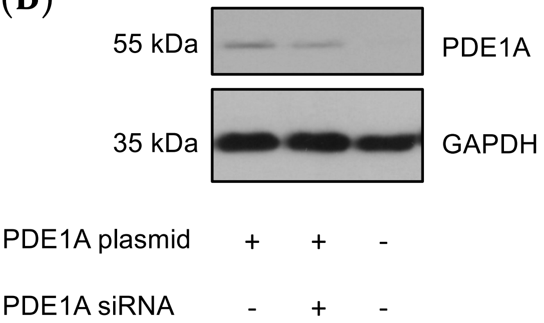

Supplementary Figure S1. Control of siRNA mediated silencing. (**A**) Isolated cardiomyocytes were transfected with control (Ctr) or PDE1A or PDE1C or PDE1A+1C siRNA for two days as indicated in Figure 7. Representative immunoblots (n=4) for PDE1C and GAPDH used as loading control. (**B**) Control of PDE1A siRNA was performed in HEK293 cells transfected with mouse PDE1A plasmid and PDE1A siRNA. Representative immunoblots (n=4) for PDE1A and GAPDH used as loading control.
